# Anti-depressive effectiveness of olanzapine, quetiapine, risperidone and ziprasidone: a pragmatic, randomized trial

**DOI:** 10.1186/1471-244X-11-145

**Published:** 2011-08-31

**Authors:** Eirik Kjelby, Hugo A Jørgensen, Rune A Kroken, Else-Marie Løberg, Erik Johnsen

**Affiliations:** 1 Division of Psychiatry, Haukeland University Hospital, Sandviken, Norway; 2 Department of Clinical Medicine, Psychiatry, University of Bergen, Norway; 3 University of Bergen, Inst. Biological and Medical Psychology, Norway

## Abstract

**Background:**

Efficacy studies indicate anti-depressive effects of at least some second generation antipsychotics (SGAs). The Bergen Psychosis Project (BPP) is a 24-month, pragmatic, industry-independent, randomized, head-to-head comparison of olanzapine, quetiapine, risperidone and ziprasidone in patients acutely admitted with psychosis. The aim of the study is to investigate whether differential anti-depressive effectiveness exists among SGAs in a clinically relevant sample of patients acutely admitted with psychosis.

**Methods:**

Adult patients acutely admitted to an emergency ward for psychosis were randomized to olanzapine, quetiapine, risperidone or ziprasidone and followed for up to 2 years. Participants were assessed repeatedly using the Positive and Negative Syndrome Scale - Depression factor (PANSS-D) and the Calgary Depression Scale for Schizophrenia (CDSS).

**Results:**

A total of 226 patients were included. A significant time-effect showing a steady decline in depressive symptoms in all medication groups was demonstrated. There were no substantial differences among the SGAs in reducing the PANSS-D score or the CDSS sum score. Separate analyses of groups with CDSS sum scores > 6 or ≤6, respectively, reflecting degree of depressive morbidity, revealed essentially identical results to the primary analyses. There was a high correlation between the PANSS-D and the CDSS sum score (r = 0.77; p < 0.01).

**Conclusions:**

There was no substantial difference in anti-depressive effectiveness among olanzapine, quetiapine, risperidone or ziprasidone in this clinically relevant sample of patients acutely admitted to hospital for symptoms of psychosis. Based on our findings we can make no recommendations concerning choice of any particular SGA for targeting symptoms of depression in a patient acutely admitted with psychosis.

**Trial Registration:**

ClinicalTrials.gov ID; URL: http://www.clinicaltrials.gov/: NCT00932529

## Background

Depressive symptoms are common in psychotic disorders, illustrated by point prevalence figures in patients with schizophrenia between 7-75% [1,2]. These figures vary due to different sub-populations and different definitions of depression. The modal rate has been estimated at 25% [2]. The identification of depression in this patient group is challenging for several reasons, including the overlap between depressive symptoms and the negative symptoms of psychosis and depressive features being common in the prodromal phase of schizophrenia [1]. Nevertheless, depression should be diagnosed and properly treated as it is associated with increased distress, poorer functional performance, a poorer quality of life, increased rates of relapse and increased mortality related to suicide [3-6].

Anti-depressive properties have been indicated for several second generation antipsychotics (SGAs) [7-11]. Different hypotheses exist regarding the mechanisms by which the SGAs mediate their anti-depressive effects, including antagonism of serotonergic 5HT_2 _receptors; agonism of 5HT_1 _receptors; antagonism of adrenergic α_2 _receptors and inhibition of trans-membrane monoamine transporters [12-14]. The evidence for efficacy is strongest in bipolar depression in which some SGAs have become agents of first choice [10]. Pragmatic studies of anti-depressive effectiveness of SGAs in more heterogeneous, naturalistic samples with psychosis are scarce [15,16]. Short-term studies do, however, indicate anti-depressive effects of several SGAs in non-affective psychosis [17]. Olanzapine was superior to haloperidol in reducing depressive symptoms in a 6-week study [18]. In patients with treatment refractory schizophrenia, quetiapine was found to be superior to haloperidol in reducing depressive symptoms during the 8-week follow-up [19]. Both studies were sponsored by the pharmaceutical industry. Some studies have indicated a marked superiority of clozapine in reducing the risk of suicide and depressive symptoms compared to the other antipsychotics [20]. In some recent studies quetiapine has demonstrated anti-depressive properties in both clinically depressed and non-depressed populations [9,21,22]. There are indications that studies sponsored by the pharmaceutical industry selectively report data in favour of the sponsored drug [23].

Clearly, more long-term studies are needed on the highly prevalent occurrence of depressive symptoms in psychosis. In particular, comparative effectiveness trials of first-line SGAs funded independently of the pharmaceutical industry are called for in order to provide clinically relevant evidence on whether or not differential anti-depressive effectiveness exists among the drugs. We have previously reported the superior effectiveness of quetiapine on several outcomes other than depression [24]. The overall depression outcome was reported only briefly. Depression and depressive symptoms are, however, the main foci in the present study with a larger sample.

The primary aim of the present pragmatic, randomized study is to investigate whether differential anti-depressive effectiveness exists among olanzapine, quetiapine, risperidone and ziprasidone, in a clinically relevant sample of patients acutely admitted to a psychiatric hospital with psychosis. The hospital is responsible for all the acute admissions in the catchment area.

## Methods

### Study design

Methods have been described in more detail in a previous publication [24]. The Bergen Psychosis Project (BPP) is a 24-month, prospective, rater-blind, pragmatic, randomized, head-to-head comparison of the effectiveness of olanzapine, quetiapine, risperidone and ziprasidone. All patients were recruited from the Division of Psychiatry at Haukeland University Hospital with a catchment population of about 400,000. The BPP was approved by the Regional Committee for Medical Research Ethics and the Norwegian Social Science Data Services. Funding of the project was initiated by the Research Council of Norway, followed by the Western Norway Regional Health Authority and Haukeland University Hospital, Division of Psychiatry. The BPP did not receive any financial or other support from the pharmaceutical industry.

### Patients

The Regional Committee for Medical Research Ethics allowed eligible patients to be included before informed consent was provided, thus entailing a clinically relevant representation in the study. Any investigation that was beyond normal clinical practice was introduced only after informed consent was obtained. Patients (age ≥ 18 years) were eligible for the study if they were admitted to the emergency ward for symptoms of psychosis as determined by a score of ≥ 4 on one or more of the following items in the Positive and Negative Syndrome Scale (PANSS): delusions, hallucinatory behavior, grandiosity, suspiciousness/persecution or unusual thought content [25] and were candidates for oral antipsychotic drug therapy. The inclusion was based on the presence of psychotic symptoms irrespective of diagnostic group, thus reflecting the diagnostic uncertainty commonly present in the early treatment phases in acutely admitted psychotic patients who are nevertheless in need of antipsychotic medication. Eligible patients met ICD-10 [26] diagnostic criteria for schizophrenia, schizoaffective disorder, acute and transient psychotic disorder, delusional disorder, drug-induced psychosis, bipolar disorder except manic psychosis and major depressive disorder with psychotic features. The diagnoses were determined by the hospital's psychiatrists or specialists in clinical psychology. Patients were excluded from the study if they: were unable to use oral antipsychotics because depot formulations were indicated, did not understand spoken Norwegian language, were candidates for electroconvulsive therapy as determined by the attending psychiatrists, were suffering from organic brain disorder - principally dementia or were medicated with clozapine on admittance. Patients suffering from manic psychosis or who, due to other behavioral or mental reasons, were unable to cooperate with the assessments were also excluded from the study. Patients with drug-induced psychoses were included only when the condition did not resolve within a few days and when antipsychotic drug therapy was indicated.

### Treatments

The pragmatic design aspired to mimic the normal clinical situation with regards to treatment allocation without compromising the randomization which protects against systematic differences between groups that are not related to the treatment. Randomization to a sequence was considered the preferred method. At admission, a sealed and numbered envelope was opened by the attending psychiatrist and then the patient was offered the first drug in a random sequence of olanzapine, quetiapine, risperidone or ziprasidone. The randomization was open to the treating psychiatrist or physician and to the patient. Both the treating clinician and/or the patient could discard the SGA listed as number 1 on the list because of medical contraindications to, or prior negative experiences with the drug. In that case the next drug on the list could be chosen. The same principle was followed throughout the sequence. A reason for discarding a drug was requested. In each sequence, the SGA listed as 1 defined the randomization group (RG). The actual SGA chosen, regardless of randomization group, defined the first-choice group (FCG). Further dosing, combination with other drugs or switching to another antipsychotic drug were then left at the clinician's discretion. Apart from sporadic use, the patients in the project could use only one antipsychotic drug, except during the cross-taper period associated with a change of antipsychotic drug. This is in correspondence with leading treatment guidelines which suggest combinations of antipsychotics be used only as a last resort [27]. In cases where concomitant use of more than one antipsychotic drug was inevitable, the patient could not participate in the project.

### Assessments

Assessments were performed at the following points of time: at baseline, at 6 weeks from baseline or at discharge if discharged before 6 weeks from baseline and at 3, 6, 12 and 24 months from baseline.

The majority of assessments were performed by one trained investigator, EJ, assisted by HAJ and RAK. Training and inter-rater reliability testing were conducted with a satisfactory inter-rater reliability. Before inclusion, eligible patients were interviewed by the investigator using the Calgary Depression Scale for Schizophrenia (CDSS) [28] and the PANSS. The CDSS has been specifically developed to assess the level of depressive symptoms in schizophrenia. Depression rating scales frequently used in mood disorders may not sufficiently distinguish depressive symptoms from positive, negative and extra-pyramidal symptoms in psychosis. The CDSS consists of 9 items, each giving a score of 0 to 3 points. The total CDSS sum score range is 0 to 27. A CDSS sum score > 6 has a specificity of 82% and a sensitivity of 85% for predicting a major depressive episode [29]. We used a cut-off of > 6 and ≤ 6 in correspondence with the guidelines from the authors of the CDSS [29]. The PANSS Depression Factor (PANSS-D) is the combined score of items G1 (somatic concerns), G2 (anxiety), G3 (guilt feelings), and G6 (depression) of the general psychopathology part of the PANSS. Each item is scored from 1 to 7, giving a total PANSS-D score ranging from 4 to 28. Several previous articles have performed factor analyses on the PANSS and described the PANSS-D as a measure of depressive symptoms in psychotic patients [30-32]. In the literature PANSS-D is also referred to as the Composite PANSS Depression Factor, PANSS Anxio-Depressive Dimension or PANSS Depression Subscale. Cognitive functioning at baseline was assessed by means of the Repeatable Battery for the Assessment of Neuropsychological Status (RBANS) [33], shown to be highly sensitive to the neurocognitive impairments associated with schizophrenia [34,35]. Misuse or dependence was reported according to Mueser et al [36].

At discharge from the hospital or at 6 weeks if not discharged, the tests and examinations were repeated by a rater who was unaware of the treatment. Serum level measurements of the antipsychotics were conducted. Thus far, all investigations and tests were part of the hospital's routine for the management of patients suffering from psychosis and became part of the patient's medical record. At this point, the patients were asked for informed consent to be contacted and included in the follow-up project.

At follow-up visits 3, 6, 12 and 24 months after baseline, measures of psychopathology were repeated by a rater blind to treatment. At each visit, all medications were recorded and the mean antipsychotic drug doses were calculated. Antipsychotic drug doses for accepted sporadic use of antipsychotics, other than the SGAs under investigation, were converted to chlorpromazine equivalent doses [37]. In cases where chlorpromazine equivalent doses could not be found in the literature, this was done by conversion to defined daily doses (DDDs) as developed by the World Health Organization Collaborating Centre for Drug Statistics Methodology [38]. The basic definition of the DDD unit is the assumed average maintenance dose per day for a drug used for its main indication in adults.

### Statistical procedures

The primary analyses were intention-to-treat (ITT) analyses based on the randomization groups (RGs). That is, trial participants were analyzed in the group to which they were randomized regardless of which treatment they actually received, or how much treatment they received [39]. Secondary analyses were based on first choice groups (FCGs). Baseline data were analyzed using SPSS software (version 17.0) and by means of exact χ^2 ^tests for categorical data and one-way ANOVAs for continuous data. For baseline comparisons between those lost to follow-up before retesting and those who were retested, independent samples T-tests were used for continuous data and exact χ^2 ^tests for categorical data.

Change of depressive symptoms was analyzed in R by means of linear mixed effects (LME) models [40,41]. Fixed effects, i.e. systematic differences between the drugs, were different linear slopes in the four treatment groups, technically a group-by-time interaction with no baseline group differences. The model calculates overall change per time unit from a common starting point for the variables in the follow-up period. This can be visually represented by the slope of a linear curve with time on the horizontal axis and the respective variable on the vertical axis. Since the aim of the present study was to investigate the overall change during the follow-up period, the LME model was considered to be the analysis of choice for this purpose. The model uses all available data and handles different numbers of visits, as well as differences in times between visits, by individual patients. Furthermore, the mixed effects model has demonstrated superior statistical power when the missing data is moderately non-ignorable [42]. For multiple comparisons, Benjamini-Hochberg adjustments were applied.

The CDSS and the PANSS are primarily developed to assess patients with schizophrenia. As the sample is diagnostically heterogeneous, a Spearman correlation analysis was performed using the SPSS software (version 17.0) to determine the consistency across the CDSS and the PANSS-D. The level of statistical significance was set at α = 0.05, two-sided.

Power estimations were conducted in R by means of LME models. The initial CDSS sum score and within-person-variation were based on the results of a previous model [24]. CDSS sum score reductions of 10%, 20%, 50% and 70% in the respective drug groups were considered to be clinically significant differences and the corresponding slopes were entered into the model. For comparison, the EUFEST study reported a 65% overall reduction of the CDSS sum score at 12 months [15]. The initial CDSS sum score was set at 5.7 points in the model and an estimated drop-out rate of 3% per month was used. For each level of power 10,000 simulations were run. Based on these premises for the power calculations, the trial should have 80% power to detect statistically significant differences among the drugs with 45 subjects in each treatment group, and 90% power with 55 subjects in each group.

## Results

**Figure 1:**
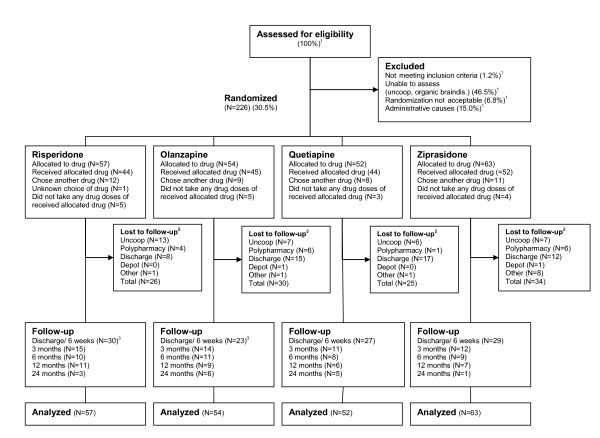
**Flow of patients through the study**. Not meeting inclusion criteria = score below 4 on all the items: delusions, hallucinatory behaviour, grandiosity, suspiciousness/persecution or unusual thought content in the Positive and Negative Syndrome Scale (PANSS); Uncoop. = the patient was not able or willing to cooperate with testing and assessments; Organic braindis. = Organic brain disorder, principally dementia; Randomization not acceptable = patient or treating clinician not willing to change existing antipsychotic medication; Administrative causes = principally patient discharged before assessments could be made. ^1 ^Enrolment started March 2003 until 2008, week 26. Full details on enrolment were only registered from 2006, week 31 until 2008, week 26. Consequently only percentages are displayed for patients assessed for eligibility and excluded patients. ^2 ^Before discharge/6 weeks. ^3 ^One patient in the risperidone and olanzapine groups missed the first follow-up visit, but was retested on later visits.

The patient enrolment is displayed in Figure 1. Baseline demographic and clinical characteristics are presented in Additional file 1. A total of 226 patients were allocated to randomized sequences of the first-line SGAs listed from 1 to 4. The SGAs listed as 1 defined the randomization groups (RGs). A total of 185 (81.9%) patients received the SGA listed as 1, whereas 40 (17.7%) received another SGA on the list. The choice of SGA was unknown for one patient. There were no differences among RGs in the fractions of patients that did not choose the SGA listed as 1. The sample represented a diverse population suffering from psychosis. Five patients were diagnosed with co-morbid major depressive disorder in addition to a primary psychotic disorder; two were in the risperidone group and one in each of the other groups.

### Primary outcomes - ITT analyses based on RGs

**Figure 2:**
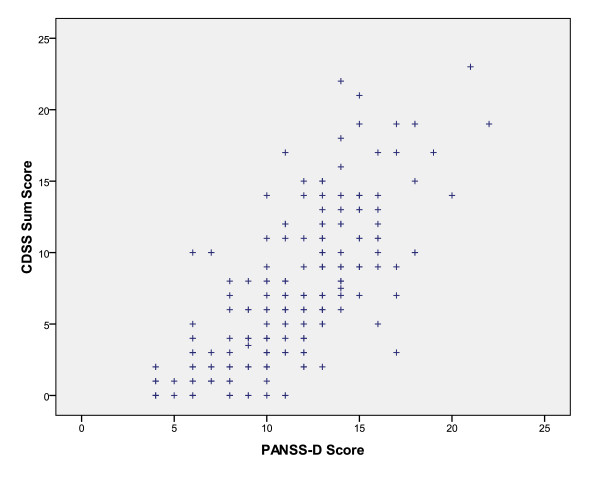
**Correlation between CDSS sum score and PANSS-D-score at baseline**. CDSS = the Calgary Depression Scale for Schizophrenia; PANSS = the Positive and Negative Syndrome Scale; PANSS-D score = PANSS Depression factor = sum score of items G1-G3 and G6 in the general psychopathology subscale of the PANSS.

**Figure 3:**
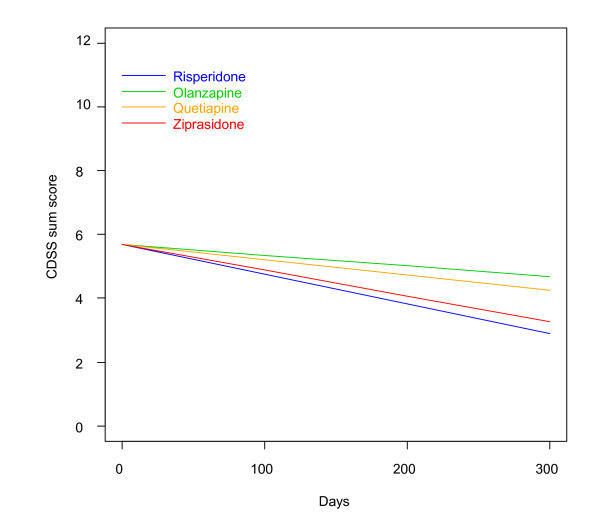
**Change of CDSS sum score**. Linear mixed effects model curves. Linear slopes for the randomization groups generated based on linear mixed effects models, CDSS sum score output, as displayed in Table 1 for olanzapine, quetiapine, risperidone and ziprasidone, respectively. The curves are confined to the first 300 days because the major bulk of data is obtained before 300 days. CDSS = the Calgary Depression Scale for Schizophrenia.

**Figure 4:**
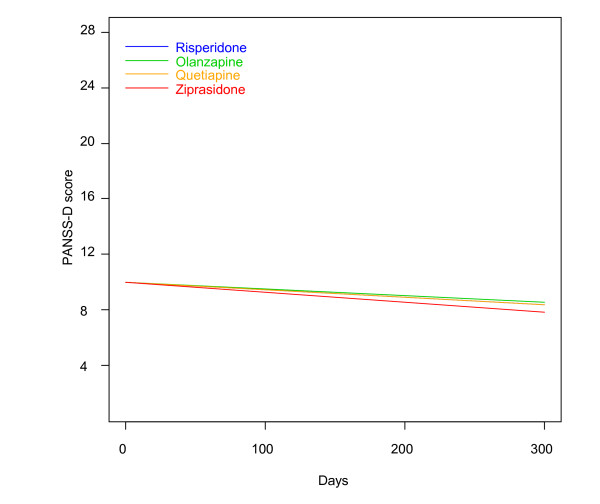
**Change of PANSS-D score**. Linear mixed effects model curves. Linear slopes for the randomization groups generated based on linear mixed effects models, PANSS-D score output, as displayed in Table 1 for olanzapine, quetiapine, risperidone and ziprasidone, respectively. The curves are confined to the first 300 days because the major bulk of data is obtained before 300 days. PANSS = The Positive and Negative Syndrome Scale; PANSS-D = PANSS Depression Factor = sum score of items G1-G3 and G6 in the general psychopathology subscale of the PANSS.

There were no statistically significant differences in baseline demographic and clinical characteristics between the RGs, thus confirming a successful randomization. There was no difference among the groups with regards to time until discontinuation of allocated drug. There were generally no substantial differences on baseline clinical or demographic characteristics between those who were lost to follow-up before retesting and those who were retested, with the exception of a slightly higher PANSS negative sub-score for those lost to follow-up (20.8 vs. 18.5 points (independent samples T-Test: p = 0.02; mean difference 2.3 points; 95% confidence interval (CI) 0.4-4.2)). The mean CDSS sum score at baseline was 6.4 points, varying between 0 to 23 points. The mean baseline PANSS-D sum score was 10.8 points, varying between 4 to 22 points. A total of 96 (42.7%) of the patients had a CDSS sum score > 6 points. The CDSS sum score and PANSS-D score correlated significantly (Spearman correlation coefficient r = 0.77; p < 0.01) (Figure 2). The symptom outcomes quantified by the CDSS and the PANSS-D are presented in Table 1. There was a significant time-effect showing a steady decline in depressive symptoms in all medication groups (Figures 3 and 4). Pair-wise comparisons demonstrated no statistically significant differences between the RGs on the primary outcomes. Analyses restricted to the first 90 days revealed no substantial differences among the SGAs. When affective psychoses and substance-induced psychoses, respectively, were excluded in sensitivity analyses for the whole follow-up, essentially the same results were revealed. In separate analyses in the groups with CDSS sum score > 6 or ≤ 6, respectively, there were no statistically significant differences between the SGAs. In sub-analyses on single CDSS items there were no statistically significant differences among the SGAs, except for item 8 (suicidality), as the risperidone group had a steeper daily reduction of the score compared to the olanzapine group (LME: p = 0.031). Corrected for multiple comparisons, this difference was no longer statistically significant (LME: p = 0.187). There were no statistically significant differences among the SGAs concerning anti-depressive effectiveness on the PANSS item G6 (depression).

**Table 1:** Numerical results of the CDSS and the PANSS-D

Outcome Measures - Change/Day	Risperidone (N = 57)	Olanzapine (N = 54)	Quetiapine (N = 52)	Ziprasidone (N = 63)
**CDSS item 1** **Depression**	-0.0016	-0.0011	-0.0017	-0.0022
**CDSS item 2** **Hopelessness**	-0.0010	-0.0002	-0.0005	-0.0008
**CDSS item 3** **Self depreciation**	0.0083	-0.0002	-0.0005	-0.0006
**CDSS item 4** **Guilty ideas of reference**	-0.0005	-0.0007	-0.0006	-0.0011
**CDSS item 5** **Pathological guilt**	-0.0010	-0.0052	-0.0004	-0.0011
**CDSS item 6** **Morning depression**	-0.0011	-0.0006	-0.0005	-0.0009
**CDSS item 7** **Early awakening**	-0.0009	-0.0002	-0.0003	-0.0003
**CDSS item 8** **Suicide**	-0.0011	-0.0002	-0.0004	-0.0006
**CDSS item 9** **Observed depression**	-0.0008	-0.0001	-0.0004	-0.0006
**CDSS sum score**	-0.0093	-0.0033	-0.0048	-0.0080
**PANSS item G6:** **Depression**	-0.0015	-0.0020	-0.0027	-0.0023
**PANSS-D score**	-0.0014	-0.0012	-0.0014	-0.0018

N = Number of Patients; Change/Day = Mean Change of Outcome Measure per Day; CDSS = The Calgary Depression Scale for Schizophrenia. PANSS = The Positive And Negative Syndrome Scale for Schizophrenia. PANSS-D = The sum score of items G1-G3 + G6 of the General Psychopathology subscale of the PANSS.

### Secondary outcomes based on FCGs

There were generally no substantial differences among FCGs on baseline demographic and clinical characteristics, with the exception of a slightly higher PANSS positive sub-score for olanzapine (21.6 points) compared with risperidone (18.4 points) (one-way ANOVA: p < 0.001; mean difference 3.2 points; 95% CI 1.1-5.3) and ziprasidone (19.2 points) (one-way ANOVA: p = 0.011; mean difference 2.5 points; 95% CI 0.4-4.5). The mean doses in milligrams per day with standard deviations (SD) were 14.5 (5.0) for olanzapine-, 339.3 (193.4) for quetiapine-, 3.3 (1.1) for risperidone- and 100.3 (42.2) for ziprasidone-treated groups. The mean serum levels in nanomoles per liter with SD were 100.4 (72.4) for olanzapine, 398.2 (510.2) for quetiapine, 81.4 (58.3) for risperidone and 122.9 (91.3) for ziprasidone. The reference ranges were 30-200, 100-800, 30-120, and 30-200 for olanzapine, quetiapine, risperidone and ziprasidone, respectively. Data concerning psychotropic treatment was analyzed for the 108 patients who were available for retesting at discharge or at 6 weeks. A total of 30 (26.5%) patients changed their first-chosen SGA during follow-up. There were no differences among the FCGs in the frequency of change or choice of new antipsychotic drug. One or more doses of low-potency first-generation antipsychotics were given to 16 patients (14.8%). There were no differences among the FCGs in the number of patients receiving additional antipsychotics or the mean daily additional antipsychotic dose in chlorpromazine equivalents. 80 (74.1%), 28 (25.9%) and 7 (6.5%) patients received additional benzodiazepines, antidepressants and mood stabilizers, respectively. In 35 (32.4%) of these patients 2 or more of the additional psychotropics were used in combination. There were no differences among FCGs in the use of these additional psychotropics. Anticholinergics were prescribed for 6 (23.1%) of risperidone-treated FCGs. The corresponding figures were 1 (3.4%) for olanzapine-, 0 for quetiapine- and 5 (18.5%) for ziprasidone-treated FCGs (exact χ^2 ^test: p = 0.009). There were no differences among FCGs in the frequency of antipsychotic drug use the year prior to index hospitalization.

The PANSS-D and CDSS scores of the primary analyses were essentially unaltered in the secondary analyses. This also applied to the sensitivity analysis restricted to the first 90 days, and to the period of actual intake of the first chosen antipsychotic drug.

## Discussion

The primary aim of the present study was to investigate whether differential anti-depressive effectiveness is present among olanzapine, quetiapine, risperidone and ziprasidone in a clinically relevant sample of patients acutely admitted to hospital for symptoms of psychosis. The study was funded independently of the pharmaceutical industry and the patients were followed for up to 2 years during everyday clinical circumstances. This strengthens the applicability of the results to acutely admitted patients with psychosis in general.

There were no substantial differences among the SGAs on the primary outcome measure. The results are in line with those of the recently published EUFEST and CATIE effectiveness studies that included schizophrenia patients in first episode and chronic phase, respectively [15,16]. The collected evidence from naturalistic studies in psychosis thus indicates that if differential anti-depressive effectiveness exists among the drugs, this is likely to be of only marginal magnitude in clinical practice. As the sample was diagnostically heterogeneous the primary outcome was measured by two different inventories: the PANSS-D and the CDSS. The correlation between the sum scores of the inventories was good, but left substantial variance unexplained, thus underlining the rationale for using both inventories in the present study. The majority of prior studies indicating anti-depressive differences among antipsychotics are short term [17,19,21,43]. If the anti-depressive effects of the drugs occur mainly within the first weeks to months of treatment, differential effectiveness may, in theory, be blurred in a longer time frame. Based on the sensitivity analyses restricted to the first 90 days of follow-up our data do not support this hypothesis. Consistent with our findings there was a significant overall decrease of the CDSS score in the CATIE study. Neither study had a placebo arm, which makes interpretation of this result difficult with regards to assessing the anti-depressive effectiveness of the drugs.

Depression is highly prevalent in first-episode and acute phase psychosis [44,45]. The mean CDSS sum score in the BPP at baseline was 6.5. The CDSS sum score was > 6 for 42.7% of the sample. DeNayer et al. [21], using the same CDSS-score cut-off as in our study, found significant reductions both in the groups with CDSS sum scores > 6 and ≤6 points, respectively. Our analyses demonstrated no statistically significant differences among the SGAs in either group based on this subdivision. The equal anti-depressive effectiveness found also in the group with CDSS sum score > 6, supports our main findings. Caution should be given to the fact that subgroup analyses increase the risk of statistical type II errors. In the CATIE study quetiapine was found to be superior to risperidone in patients with a CDSS score ≥6 [16]. However, the more depressed group became relatively larger in the CATIE-trial as a consequence of the lower cut-off (CDSS ≥ 6) for depression.

The sample was diagnostically heterogeneous, though with equal diagnostic distribution among the RGs. Hypothetically, different diagnostic groups could differ in anti-depressive susceptibility from the SGAs, which could blur the overall picture. We therefore conducted sensitivity analyses, excluding the affective psychoses and substance-induced psychoses which did not skew the results. The proportion of primary affective disorders was rather low.

Some limitations apply to the study. In a moderately sized clinical trial like the Bergen Psychosis Project, the possibility of a type II statistical error exists. The BPP has, however, proven statistically powerful enough to disclose differences among the SGAs on several outcomes [24]. Furthermore, power analyses indicate that the study should have a sufficient number of subjects to detect clinically significant differences in anti-depressive effectiveness among the drugs, if present. The pragmatic design was chosen to address issues relevant to everyday clinical practice. The resulting heterogeneous sample does not have enough power to conclude statistically inside particular diagnostic subgroups. The randomization procedure allowing the patient or clinician to choose a different drug than the first one could potentially introduce bias if there were differences among the groups in the proportions accepting the first SGA on the list. No such differences were unveiled. Furthermore, the primary analyses were intention to treat analyses based on the randomization groups. It could be argued given the naturalistic design of the study, with assessments not restricted to the time frame of actual use of the first SGA, that the outcomes may not be related to that particular SGA, but to subsequent medications. We have, however, demonstrated that about three-quarters of the patients did not change their original SGA. Moreover, there were no differences among groups in the rate of antipsychotic medication changes or the choice of a new antipsychotic agent for those who did change. Furthermore, time until discontinuation was generally the same for all SGAs. Finally, the analyses restricted to the period of actual use of first chosen drug revealed generally the same results as the primary analyses. Inherent to the pragmatic design which permits the use of concomitant psychotropics, the net effects of the SGAs under investigation may be somewhat blurred by effects of the concomitant psychotropics. The randomization was open to the patient and the treating clinician in order to imitate a clinically realistic setting. This could have introduced bias if some of the SGAs were more popular among the clinicians or patients. There was a high attrition rate, although not significantly different between the randomization groups. To our best knowledge this is a major problem in all clinical antipsychotic drug trials. Leucht and collaborators [46] state in their methodology paper that even in short-term trials of only 4 to 10 weeks more than 40% of participants discontinue prematurely. Given the long follow-up of our study we expected a high drop-out rate. This was the main reason for the choice of the mixed-effects statistical method applied, as this method is one of the preferred ones in such a situation. Reasons for drop-out were not recorded and the possibility that the more depressed patients dropped out cannot be ruled out. Still, comparisons on baseline characteristics between those with long term follow-ups and the ones leaving the study early, do not point to substantial clinical differences among the groups. Of those assessed for eligibility, only 30% were included in the trial. Theoretically, including only a fraction of eligible participants could limit the applicability of the results to the whole population. On the other hand, other clinical trials studying antipsychotics included only between 7-14% [47]. The diagnoses were determined by psychiatrists or specialists in clinical psychology, and structured clinical interviews were not systematically used, which may decrease the validity and reliability of the diagnoses. The ITT-analyses may lead to an underestimation of treatment effect. However, the substantially equal results of the ITT- and FCG-analyses indicate that this was not the case in this trial. Analyses involving single items in the CDSS should be interpreted with caution as the data are unlikely to be normally distributed. Finally, the CDSS-instrument is designed to measure depressive symptoms in schizophrenia specifically. Our sample was diagnostically heterogeneous. Somewhat surprisingly, considering that few trials indicate a superior antipsychotic effectiveness of quetiapine [15,48,49], the recently published results of the Bergen Psychosis Project demonstrated a significant superiority of quetiapine compared with olanzapine and risperidone on several psychometric scales. The present study shows that the superiority of quetiapine could not be explained by a stronger anti-depressive effect.

## Conclusions

In conclusion, the results of this study demonstrate no substantial differences in anti-depressive effectiveness between olanzapine, quetiapine, risperidone and ziprasidone in a clinically relevant sample of psychotic patients with moderate depressive symptoms. Based on our findings we can make no recommendations concerning choice of any particular SGA for targeting symptoms of depression in a patient acutely admitted with psychosis.

## Competing interests

Funding of the project was initiated by the Research Council of Norway, followed by Haukeland University Hospital, Division of Psychiatry. The supporters had no role in the design and conduct of the study; collection, management, analysis, and interpretation of the data; or preparation, review or approval of the manuscript.

Kjelby E has been reimbursed by Bristol-Myers Squibb, Novartis, Lundbeck, Eli Lilly and AstraZeneca pharmaceutical companies for attending conferences.

Jørgensen HA has received honoraria for lectures given in meetings arranged by AstraZeneca and Eli Lilly.

Kroken R has been reimbursed by the Eli Lilly Company, Janssen Cilag Company, Bristol-Myers Squibb and AstraZeneca for attending conferences.

Løberg EM has no competing interests to declare.

Johnsen E has received honoraria for lectures given in meetings arranged by Bristol-Myers Squibb, Eli Lilly, and AstraZeneca, and for a contribution to an information brochure by Eli Lilly. Erik Johnsen has been reimbursed by the Eli Lilly Company and the Janssen Cilag Company for attending conferences.

## Authors' contributions

EK drafted the manuscript and participated in the statistical analyses. EJ helped to draft the manuscript, provided statistical analyses and performed the main part of the data collection. EML, RAK and HAJ helped to draft the manuscript and participated in the data collection. All authors read and approved the final manuscript.

## Pre-publication history

The pre-publication history for this paper can be accessed here:

http://www.biomedcentral.com/1471-244X/11/145/prepub

## Supplementary Material

Additional file 1 **Demographic and clinical characteristics at baseline**. This table displays baseline comparisons of demographic characteristics, clinical characteristics (drug- and alcohol-use, diagnoses, antipsychotic-naïve) and baseline psychometric results between the randomization groups. Click here for file
