# Supplementary material for: Anti-depressive effectiveness of olanzapine, quetiapine, risperidone and ziprasidone: a pragmatic, randomized trial
Source: BMC Psychiatry. 2011 Aug 31;11:145. doi: 10.1186/1471-244X-11-145 (PMC3178484; doi:10.1186/1471-244X-11-145)
Supplement: Additional file 1 — Demographic and clinical characteristics at baseline. This table displays baseline comparisons of demographic characteristics, clinical characteristics (drug- and alcohol-use, diagnoses, antipsychotic-naïve) and baseline psychometric results between the randomization groups. [file 1471-244X-11-145-S1.DOC]

Additional file 1: Demographic and clinical characteristics at baseline.

|  | | Randomization Groups | | | | | | | | |  | |
| --- | --- | --- | --- | --- | --- | --- | --- | --- | --- | --- | --- | --- |
| Characteristics | | Risperidone (N=57) | | Olanzapine (N=54) | | Quetiapine (N=52) | | Ziprasidone (N=63) | | All Patients(N=226) | | |
|  | | N | % | N | % | N | % | N | % | N | | % |
| Gender | |  |  |  |  |  |  |  |  |  | |  |
|  | Male | 41 | 71.9 | 34 | 63.0 | 35 | 67.3 | 42 | 66.7 | 152 | | 67.3 |
| Ethnicity | |  |  |  |  |  |  |  |  |  | |  |
|  | White | 50 | 87.7 | 49 | 90.7 | 49 | 94.2 | 57 | 90.5 | 205 | | 90.7 |
|  | Other | 7 | 12.3 | 5 | 9.3 | 3 | 5.8 | 6 | 9.5 | 21 | | 9.3 |
| Antipsychotic naive | | 21 | 37.5 | 20 | 37.0 | 28 | 53.8 | 30 | 48.4 | 99 | | 44.2 |
|  | |  |  |  |  |  |  |  |  |  | |  |
| Alcohol last 6 mths | |  |  |  |  |  |  |  |  |  | |  |
|  | None | 16 | 28.1 | 10 | 18.5 | 9 | 17.3 | 12 | 19.4 | 47 | | 20.8 |
|  | Misuse | 3 | 5.3 | 5 | 9.3 | 10 | 19.2 | 5 | 8.1 | 23 | | 10.2 |
| Drugs last 6 mths | |  |  |  |  |  |  |  |  |  | |  |
|  | None | 32 | 61.5 | 38 | 73.1 | 36 | 72.0 | 38 | 66.7 | 144 | | 68.2 |
|  | Misuse | 11 | 21.2 | 9 | 17.3 | 7 | 14.0 | 11 | 19.3 | 38 | | 18.0 |
| Diagnosis^1^ | |  |  |  |  |  |  |  |  |  | |  |
|  | Schz and rel. | 27 | 50.9 | 20 | 38.4 | 23 | 44.2 | 26 | 44.1 | 96 | | 44.4 |
|  | Acute | 7 | 13.2 | 15 | 28.8 | 12 | 23.1 | 12 | 20.3 | 46 | | 21.3 |
|  | Drug-induced | 10 | 18.9 | 6 | 11.5 | 7 | 13.5 | 6 | 10.2 | 29 | | 13.4 |
|  | Affective | 4 | 7.5 | 6 | 11.5 | 7 | 13.5 | 6 | 10.2 | 23 | | 10.6 |
|  | Rest | 5 | 9.4 | 5 | 9.6 | 3 | 5.8 | 9 | 15.3 | 22 | | 10.2 |
|  |  | Mean | SD/Range | Mean | SD/Range | Mean | SD/Range | Mean | SD/Range | Mean | | SD |
| Age | | 33.8 | 13.0/ 18-67 | 31.8 | 12.1/ 18-72 | 36.9 | 13.7/ 18-72 | 33.9 | 14.6/ 17-73 | 34.1 | | 13.5/17-73 |
| PANSS Total | | 74.3 | 12.9/ 51-110 | 75.1 | 14.0/ 44-111 | 74.8 | 14.3/ 47-111 | 72.2 | 12.7/ 45-109 | 74.0 | | 13.4/ 44-111 |
| PANSS Positive | | 18.8 | 4.5/ 12-32 | 20.8 | 4.5/ 11-32 | 20.2 | 4.0/ 12-31 | 19.6 | 4.5/ 11-31 | 19.8 | | 4.4/ 11-32 |
| PANSS Negative | | 21.5 | 7.5/ 7-39 | 18.5 | 7.7/ 7-38 | 19.9 | 7.1/ 7-33 | 18.9 | 7.1/ 8-37 | 19.7 | | 7.4/ 7-39 |
| PANSS General | | 34.0 | 6.2/ 20-51 | 35.8 | 7.4/ 20-54 | 34.8 | 7.4/ 23-56 | 33.7 | 6.3/ 21-53 | 34.5 | | 6.8/ 20-56 |
| CDSS | | 6.7 | 5.7/ 0-19 | 6.5 | 4.7/ 0-19 | 6.4 | 4.6/ 0-19 | 6.4 | 6.0/ 0-23 | 6.5 | | 5.3/ 0-23 |
| GAF-F | | 31.0 | 5.3/ 18-55 | 30.6 | 5.3/ 10-41 | 30.1 | 6.7/ 15-62 | 30.9 | 6.6/ 2-45 | 30.7 | | 6.0/ 2-62 |
| CGI | | 5.2 | 0.6/ 4-6 | 5.2 | 0.7/ 4-6 | 5.2 | 0.6/ 4-6 | 5.0 | 0.6/ 4-6 | 5.2 | | 0.6/ 4-6 |
| Cognition, t-scores | | 40.1 | 7.7/ 23.0-58.8 | 38.0 | 7.8/ 20.2-52.4 | 36.3 | 7.0/ 23.8-50.6 | 38.4 | 8.0/ 21.4-55.2 | 38.2 | | 7.7/ 20.2-58.8 |

Notes:

N = number of patients; SD = standard deviation; Antipsychotic naïve = No life-time exposure to antipsychotic drugs before index admission; First admission = Index admission is the first admission to a mental hospital; Misuse = Misuse or Dependence according to Mueser et al [36]; Schz and rel. = Schizophrenia and related disorders: Schizophrenia, schizo-affective disorder, a****cute polymorphic psychotic disorder with symptoms of schizophrenia, acute schizophrenia-like psychotic disorder,**** delusional disorder; Acute = Acute psychosis other than those categorized under Schz and rel.; Affective = Affective psychosis; Rest = Miscellaneous psychotic disorders. All diagnoses are according to ICD-10; PANSS = the Positive and Negative Syndrome Scale; CDSS = the Calgary Depression Scale for Schizophrenia; GAF-F = the Global Assessment of Functioning, split version, Functions scale; CGI = the Clinical Global Impression, severity of illness scale; Cognition t-scores = Mean t-scores on the RBANS (Repeatable Battery for the Assessment of Neuropsychological Status)

^1^ Patients with missing diagnoses are not included in list.
